# Systematic Review with Network Meta-Analysis: Efficacy of Induction Therapy with a Second Biological Agent in Anti-TNF-Experienced Crohn's Disease Patients

**DOI:** 10.1155/2018/6317057

**Published:** 2018-07-17

**Authors:** Cristiano Pagnini, Spyros I. Siakavellas, Giorgos Bamias

**Affiliations:** ^1^Digestive and Liver Disease Department, S. Andrea Hospital, Rome, Italy; ^2^Academic Department of Gastroenterology, Laiko General Hospital, University of Athens Medical School, Athens, Greece; ^3^Gastroenterology Unit, Third Depatment of Internal Medicine, Sotiria General Hospital, University of Athens Medical School, Athens, Greece

## Abstract

**Background and Aim:**

Crohn's disease (CD) is a chronic inflammatory condition of the gastrointestinal tract with the potential to progress to a severe debilitating state. Treatment with biological agents is highly efficient, improving both short-term outcomes and long-term prognosis. Nonetheless, up to 60% of patients receiving biological therapy will exhibit nonresponse at some point. The optimal management of such patients is not clearly defined. Besides traditional anti-TNF agents (infliximab, adalimumab, and certolizumab), alternative biological therapies (natalizumab, vedolizumab, and ustekinumab) are currently available for the treatment of CD. Our aim was to analyze all available evidence regarding efficacy of a second biological in “biological-treatment-experienced” patients.

**Methods:**

A systematic review of the literature was conducted using specific criteria for selecting relevant randomized clinical trials evaluating response to administration of secondary biological therapy in “anti-TNF-experienced” CD patients. Data from these studies was used to perform (a) traditional meta-analysis to ascertain the effect of secondary treatment versus placebo and (b) network meta-analysis to compare indirectly the efficacy of available biological agents.

**Results:**

Out of initially 977 studies, only eight were included for analysis, providing a total of 1281 treated and 733 placebo-receiving CD patients. Treatment with a second biological was found to be superior to placebo for both induction of remission (OR 2.2, 95% CI 1.7 to 3) and response (OR 1.9, 95% CI 1.5 to 2.5), with global rates of 24% and 42%, respectively (placebo rate: 11% and 27%, *p* < 0.0001 for both). Indirect comparisons performed with network meta-analysis suggest no specific agent is clearly superior to others, with relatively better results observed for adalimumab in inducing disease remission.

**Conclusion:**

In anti-TNF-experienced CD patients, secondary biological administration may be efficient, while no specific agent seems to outperform the others. Further research is needed to identify optimal management strategies for this challenging subset of patients.

## 1. Introduction

Crohn's disease (CD) is a pathologic condition of unknown etiology that is characterized by dysregulated mucosal immunity, resulting in recurrent-remittent inflammation of the bowel [1]. Its clinical course consists of an early inflammatory phenotype, which tends to progress towards stenotic or fistulizing complications, thus resulting in a gradually debilitating disease [2]. In line with this, it has been suggested that potent and aggressive treatment interventions during the early phase of CD may hopefully arrest disease progression and preclude a degenerative course. Such favorable outcomes have been possible in recent years with the development and application of biological agents in the treatment of CD. Those target-specific immunological therapies have shown significant efficacy in the induction and maintenance of remission in CD patients. In fact, they may affect not only short-term outcomes (i.e., symptoms relief, corticosteroids withdrawal, and mucosal healing) but also long-term disease prognosis (hospitalization, surgery, and quality of life) [3, 4].

Monoclonal antibodies that neutralize TNF were pioneer drugs and have been the first biological agents that were used in CD. Those include the recombinant chimeric human-murine antibody infliximab and the recombinant human antibody adalimumab which are marketed in US and Europe for almost two decades. A third antibody, the recombinant humanized Fab' fragment certolizumab pegol, is currently approved only by the FDA. Due to their well-established efficacy, anti-TNF antibodies have been increasingly chosen as rescue therapies for conventional-treatment-refractory CD as well as early treatment for CD patients with features of severe disease and/or prognostic factors for poor prognosis [5, 6]. Notwithstanding their aforementioned efficacy, almost one third of patients will not respond ab initio (primary failure), and probably more than one-third exhibit loss of response during the next months/years (secondary failure) or may not be able to tolerate this particular treatment regimen [7–9]. Therefore, about 60% of patients who start anti-TNF therapy will be classified as “treatment-failures” at some point, representing a particularly challenging subgroup of patients for future treatment. Considering the increasing use of anti-TNF drugs and the fact that for many years anti-TNF inhibitors have been the only available option for biological treatment (except for natalizumab that had been of limited availability [10]), it is easy to infer that the number of the patients in this subgroup of anti-TNF-experienced patients is rapidly increasing, and guidance about the management of such patients is urgently needed.

In the last few years, novel molecules have become available with mechanisms of action different from anti-TNF blockade, and many more are tested in the therapeutic pipeline for CD [11]. Consequently, therapeutic options for anti-TNF-experienced CD patients are constantly expanding, creating new opportunities for the management of those difficult-to-treat patients. Biological drugs with alternative mechanism of action currently available include natalizumab (a recombinant humanized monoclonal IgG4 antibody against the integrin subunit *α*4, which, as already mentioned, is marketed only in US), vedolizumab (a humanized IgG1 monoclonal antibody blocking the enteric-specific *α*4*β*7 integrin), and ustekinumab (a fully human IgG1 monoclonal antibody against the p40 subunit that is common in the IL-12 and IL-23 molecules) [10, 12–18].

The aim of the present systematic review is to analyze all available evidence from randomized controlled trials (RCTs) in literature regarding the efficacy for induction of remission or response of the aforementioned biological agents in CD patients who had already had a previous biologic treatment (“biological treatment-experienced patients”). To accomplish our aim, we performed a traditional meta-analysis to evaluate the effect of a second biological treatment versus placebo and a network meta-analysis to indirectly compare available drugs, since no head-to-head comparisons have been conducted.

## 2. Methods

### 2.1. Data Sources and Search

The study was conducted in accordance with the PRISMA statement [19]. A search of clinical trials in PubMed and Cochrane Library databases was performed using the following search strategy: “adalimumab OR infliximab OR certolizumab OR vedolizumab OR natalizumab OR ustekinumab AND crohn.” The studies of interest were placebo-controlled, randomized studies; retrospective and observational studies were not included in any of the analyses. The search was not limited by language, but a large majority of the manuscripts were originally published in English.

### 2.2. Study Selection

Two investigators independently reviewed the titles of all identified citations to generate a list of potentially relevant articles for further review. The abstracts of these articles were reviewed to identify studies suitable for inclusion in our final analyses. For a manuscript to be eligible for our study, it had to satisfy the following eligibility criteria: (i) studies had to examine the effect of a single biological agent on induction of response or remission in CD; (ii) studies had to include in the analysis patients who had already received previously treatment with an anti-TNF agent; (iii) studies could not duplicate data already published; (iv) studies needed to be published as full manuscripts; (v) response or remission had to be defined by standardized quantitative scoring criteria (typically using the Crohn's Disease Activity Index (CDAI)); (vi) studies had to be placebo-controlled, randomized clinical trials with treatment and control arms. We did not include nonrandomized controlled trials given the concern for study heterogeneity.

### 2.3. Data Synthesis and Analysis

The odds ratio (OR) was used to measure treatment effects in all comparisons. Study-level ORs with 95% confidence intervals (CIs) were calculated in accordance with the intention-to-treat principle.

The meta-analysis was performed using the Mantel-Haenszel method calculating the fixed and random effect when indicated. Heterogeneity between studies was calculated by *χ*^2^ test. Software MedCalc version 9.2 was used for statistical calculations.

For network meta-analysis, a frequentist approach was used with the graphical tools developed by Chaimani et al. for the STATA software [20]. In short, multivariate random-effects meta-analyses were fit to model the intervention effects across studies. Effect sizes for network meta-analysis were described with 95% credible intervals. The off-diagonal cells of the league tables calculated contain odds ratios and 95% confidence intervals from all pairwise comparisons in network meta-analyses. The contribution of direct evidence to the mixed estimates and the entire network was also calculated and plotted. Probabilities of each treatment being at a specific order and surface under the cumulative ranking area (SUCRA) values were estimated running 10,000 replications. SUCRA is used to provide a hierarchy of treatments for each outcome. The larger the SUCRA value was, the better the treatment was considered. STATA 13 MP software was used to conduct the network meta-analysis.

All *p* values are 2-tailed. For all tests (except for heterogeneity), a *p* < 0.05 indicates statistical significance.

## 3. Results

### 3.1. Search Results

**Figure 1:**
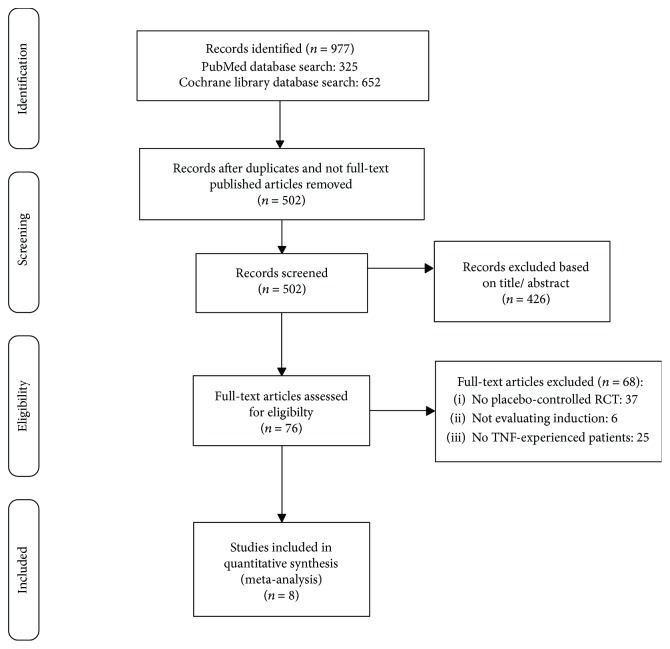
Summary of evidence search and selection for induction of response and remission in anti-TNF-experienced CD patients.

A flow diagram combining the search and selection process is presented in Figure 1. Initial literature search for clinical trials yielded a total of 977 citations, among which 325 from PubMed database and 652 from Cochrane library database. After exclusion of duplicates and nonfull-text published articles (i.e., conference proceedings and abstract of congress presentations), 502 studies were reviewed. Among those, 426 records were excluded after title and/or abstract evaluation, and the remaining 76 studies were evaluated in full-text. Sixty-eight studies were further excluded for the following reasons: not being placebo-controlled RCT (*n* = 37), not evaluating induction of remission (*n* = 6), and not including anti-TNF-experienced CD patients (*n* = 25). Finally, 8 studies [21–28] were included in the meta-analysis, with a total of 1281 treated and 733 placebo CD patients. Among those, 5 studies evaluated both remission and response, 2 only response and 1 only remission. In 4 studies, TNF-experienced patients represented the whole population analyzed, while in 4 studies, they represented a subgroup of patients. Characteristics of the 8 studies that fulfilled inclusion criteria are summarized in Table 1.

### 3.2. Direct Treatment Comparison (Treatment versus Placebo)

**Figure 2:**
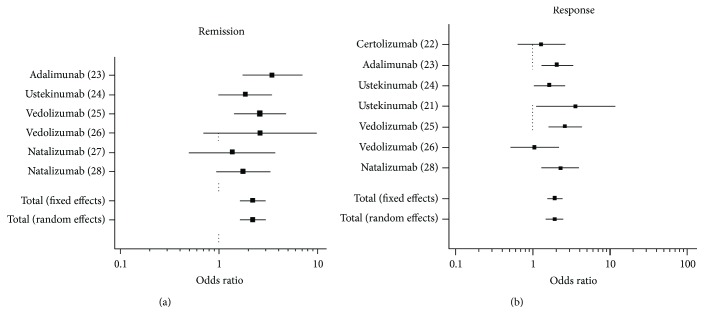
Traditional meta-analysis of published studies for induction of remission (a) and response (b) in anti-TNF-experienced CD patients. The name of the drug and the reference of the study is reported. Global comparison showed significant efficacy of treatment comparing with placebo.

Results of traditional meta-analysis showed that treatment with a second biologic drug in anti-TNF-experienced CD patients was superior to placebo in inducing remission (OR 2.2, 95% CI 1.7 to 3) and response (OR 1.9, 95% CI 1.5 to 2.5) (Figures 2 and 2). Globally, 273/1159 (24%) of patients in the treatment group achieved remission comparing with 71/621 (11%) in the placebo group. In addition, clinical response was achieved by 519/1229 (42%) versus 192/706 (27%), respectively, (*p* < 0.001 for both; *χ*^2^ test). Heterogeneity across studies was not significant (*p* = 0.59 and *p* = 0.26 for remission and response, resp.).

### 3.3. Indirect Treatment Comparison (Treatment versus Treatment)

**Figure 3:**
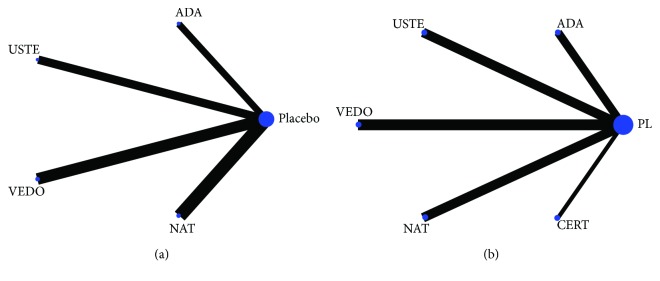
Network diagrams of published studies for induction of remission (a) and response (b) in anti-TNF-experienced CD patients. In brief, circles correspond to each intervention as a node while lines represent the direct comparisons. The size of each circle indicates the number of included participants for each corresponding treatment, and line thickness indicates the number of studies included in each comparison. Placebo as expected is represented by the biggest node. ADA = adalimumab; USTE = ustekinumab; VEDO = vedolizumab; NAT = natalizumab; CERT = certolizumab; PL = placebo.

For the network meta-analysis, initially, the network diagrams were generated separately for the two outcomes of the included studies (i.e., remission and response after induction of secondary biological treatment) as shown in Figure 3. No direct comparisons were made between therapeutic regimens, so comparisons were performed in a pairwise manner as described above. Due to the criteria of our study design (only RCTs with two arms, the one being placebo administration), no inconsistency was observed in our model while calculated heterogeneity values were not significant.

**Figure 4:**
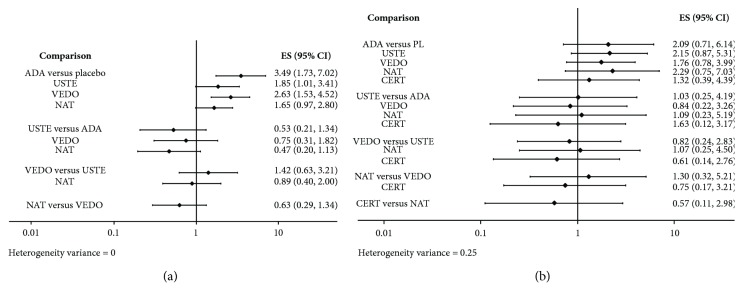
Network meta-analysis interval plot of published studies for induction of (a) remission and (b) response in anti-TNF-experienced CD patients. Odds ratios (ORs) with 95% confidence interval (CI) are reported. All the single drugs showed consistent efficacy comparing with placebo while no drug proved to be superior in a statistically significant level to all others in inducing remission. ADA = adalimumab; USTE = ustekinumab; VEDO = vedolizumab; NAT = natalizumab; CERT = certolizumab; PL = placebo.

Next, interval plots were calculated (relative efficacy is plotted as OR with 95% credible intervals). These plots demonstrate that no secondary biological agent was superior to others in a statistically significant level in inducing remission or response (Figure 4). Interestingly, our results indicate that the statistically significant superiority of biological agents in comparison to placebo persists in the network meta-analysis for remission but not for response.

**Figure 5:**
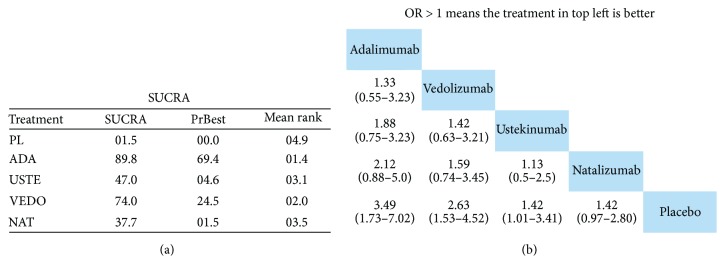
Network meta-analysis and ranking of treatments for inducing remission in anti-TNF-experienced CD patients via (a) SUCRA analysis and (b) league table graph. Odds ratios (ORs) with 95% confidence interval (CI) are reported. ADA = adalimumab; USTE = ustekinumab; VEDO = vedolizumab; NAT = natalizumab; PL = placebo.

**Figure 6:**
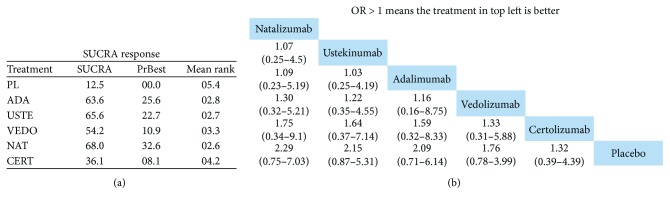
Network meta-analysis and ranking of treatments for inducing response in anti-TNF-experienced CD patients via (a) SUCRA analysis and (b) league table graph. Odds ratios (ORs) with 95% confidence interval (CI) are reported. ADA = adalimumab; USTE = ustekinumab; VEDO = vedolizumab; NAT = natalizumab; PL = placebo.

Furthermore, so as to rank treatments, a SUCRA (i.e., the converted value reflecting the probability of a treatment being the best according to the ranking of each treatment) analysis was conducted and league tables were generated. Our calculations indicate that adalimumab showed relatively better efficacy in inducing remission than the other drugs followed up in second place by vedolizumab (Figure 5). Nevertheless, regarding the achievement of clinical response, the results were more ambivalent; natalizumab, ustekinumab, and adalimumab produced similar SUCRA values that were relatively better from vedolizumab and certolizumab (Figure 6).

## 4. Discussion

The utilization of anti-TNF monoclonal antibodies for the treatment of CD for the last 20 years has resulted in a substantial, anti-TNF-experienced population of patients. Included therein are patients that failed to respond, those who lost their initial response, and those who were forced to discontinue therapy due to toxicity concerns. Our present meta-analysis clearly demonstrates that there is an increasing number of choices for such anti-TNF failures, which include a second anti-TNF monoclonal antibody, the anti-integrins vedolizumab and natalizumab, and, lastly, the anti-IL-12/IL-23 antibody ustekinumab. According to our network meta-analysis, no single treatment appears to be superior in indirect comparison.

The efficacy of biologic treatment has been highlighted by different RCTs and recently summarized in a meta-analysis by Stidham et al. [29] that analyzed in a network meta-analysis 10 studies evaluating efficacy of anti-TNF agents in induction and maintenance of remission and response in CD patients, irrespectively of their prior exposure to anti-TNF therapy. They reported that biological agents exhibited consistently superiority versus placebo. On the other hand, no clear evidence of clinical superiority of one drug versus the others could be elicited. Another research group restricted their analysis to efficacy of biologic drugs in TNF naïve patients, finding 17 studies which evaluated 6 different biologic agents. The authors came to the conclusion that infliximab was found to surpass other agents in achieving remission in this group of patients [30]. To date, the only study specifically evaluating efficacy of biologic drugs in anti-TNF-experienced patients with CD is a recent meta-analysis by Gisbert et al. [7]. In this study, authors included not only RCTs but also case-controlled and retrospective studies. Their findings mainly focused on the cause of previous anti-TNF failure as the main factor determining response to a secondary biological agent. Thus, it was suggested that patients who had a primary nonresponse had a lower remission and response rate to a second anti-TNF, while patients with loss of response or intolerance to the first anti-TNF had higher rates of success. In accordance with that observation, the only two studies included in the present work who analyzed administration of a second anti-TNF (namely adalimumab [23] and certolizumab [22]) excluded patients with primary nonresponse. This is in line with the currently prevailing opinion that recommends utilization of a biological with a different mechanism of action (switch out of class) in such patients [8].

Our study included only RCTs and evaluated specifically anti-TNF-experienced CD patients. As shown by the traditional meta-analysis section, secondary biological treatment was significantly superior to placebo for both induction of remission and response. Since no head-to-head study directly comparing two biologic drugs is currently available, we indirectly compared secondary biologicals with one another by means of network meta-analysis. Our analysis based on a frequentist approach implies that in this patient population, no single drug clearly outcompetes the other candidates in achieving remission/response. Further studies, with a prospective design and with different biological-treatment arms (including novel agents), will answer the question of which medication best suits patients failing anti-TNF therapy. Moreover, prospective studies that will evaluate not only the effect of anti-TNF but also of anti-integrin and anti-IL12/23 administration in the future course of biologically naïve CD patients are warranted to elucidate optimal management of nonresponse or treatment-intolerant patients and open the way for highly effective personalized therapeutic interventions. Until such studies become available, the choice of drug needs to be empirically tailored to the characteristics of each case, as well as local availability and patient preference issues. Interestingly, analysis of the included studies clearly shows that a considerable proportion of these patients will not respond to a secondary biological intervention. Identification of clinical, genetic, and/or molecular characteristics that may predict a beneficial response to each drug is urgently needed for these “difficult-to-treat” patients.

We are aware that the present study has several limitations. First, in the included studies, TNF-experienced patients represented either the whole population, and this had been a specific consideration in the inclusion criteria (in four studies) or a part of the population included in which a subgroup analysis was performed (in four studies). This disparity could imply a degree of selection bias that has to be taken into account. Nevertheless, when analyzing separately these two categories of studies, only a trend was observed towards higher response rate in studies that included only anti-TNF-experienced patients versus studies that included anti-TNF naïve patients as well (OR: 2.4 (95% CI: 1.7–3.3) versus 1.9 (1.1–3.3) and 2.1 (1.6–2.7) versus 1.7 (1.1–2.8), for response and remission, respectively).

Second, the reason for discontinuation of the first anti-TNF or the precise timeframe and the duration of the first biological treatment was not always specified in the included trials. Considering that anti-TNF primary failure patients have been excluded from the adalimumab and certolizumab trials, a degree of heterogeneity is to be expected regarding the cause of discontinuation in the included studies. This in turn may have affected the response/remission rate across different studies. A case, for instance, could be made that patients with a primary nonresponse may represent a subgroup with a more “difficult to treat” disease. As a result, studies that exclude or enroll a low number of such patients may be more likely to have better results. This may partially explain the trend for better results observed for adalimumab in the network comparison. On the other hand, while this same subgroup of patients was excluded in the certolizumab trial as well, no similar improved efficacy was noted.

In view of the aforementioned known problems, we decided to include only RCTs and excluded case-controlled and retrospective studies in order to reduce heterogeneity. We also chose to examine as outcomes only the achievement of response and/or remission in these patients to a second-line biological treatment, as the definitions for these particular outcomes were comparable among different trials. Moreover, inclusion criteria regarding severity and characteristics of baseline disease were also found to be quite similar across the studies we analyzed. This approach resulted to the fact that heterogeneity among studies was shown to be not significant in our meta-analysis. Nonetheless, minor differences still exist, that is, in the evaluation time points (that ranged from four to ten weeks) and in drug administration protocols, that may confound our comparisons. Apart from that, the strict criteria we chose for inclusion in our review while helpful in limiting heterogeneity led to a relatively small number (*n* = 8) of studies with a total of 2014 anti-TNF-experienced patients (1281 receiving secondary biological treatment and 733 placebo) being selected, which may limit the strength of our analysis.

In conclusion, anti-TNF-experienced CD patients requiring second-line biological therapy constitute an important and constantly expanding clinical conundrum, as management strategies are not always clear. Our systematic review, including a well-executed traditional and network meta-analysis, demonstrates that in these cases, administration of second-line biological treatment results in significant efficacy regarding remission and response when compared to placebo but with no evidence of superiority for a specific therapeutic regimen. Although such findings are clearly encouraging, still an improved characterization and classification of CD patients is needed with the aid of relevant molecular biomarkers or clinical features in order to appropriately identify disease phenotypes with specific responses to selected therapeutic regimens. Our end goal should be a highly tailored therapy with improved efficacy and outcomes in all our patients, minimizing unnecessary drug exposure and adverse events.

## Figures and Tables

**Table 1:** Characteristics of the studies included in the meta-analysis.

Study (ref)	Drug	*N* drug/placebo	Inclusion	Outcome	Population characteristic	Time (wks)
Sandborn et al., PRECISE 1 [22]	Certolizumab	97/85	No primary nonresponders	Response	Subgroup	6
Sandborn et al., GAIN [23]	Adalimumab	159/166	No primary nonresponders	Remission/response	Whole	4
Sandborn et al. [24]	Ustekinumab	394/132	Primary/secondary nonresponse, adverse events	Remission/response (3 different doses)	Whole	8
Sandborn et al. [21]	Ustekinumab	25/27	Previous exposure	Response (2 different doses)	Subgroup	8
Sands, GEMINI 3 (25)	Vedolizumab	158/157	Primary/secondary nonresponse, adverse events	Remission/response	Whole	10
Sandborn et al. [26]	Vedolizumab	105/70	Primary/secondary nonresponse, adverse events	Remission/response	Subgroup	6
Sands et al. [27]	Natalizumab	52/27	Secondary nonresponders	Remission	Whole	10
Sandborn et al. [28]	Natalizumab	291/69	Not specified	Remission/response	Subgroup	10
